# Implication of the Type III Effector RipS1 in the Cool-Virulence of *Ralstonia solanacearum* Strain UW551

**DOI:** 10.3389/fpls.2021.705717

**Published:** 2021-07-22

**Authors:** Jeffrey K. Schachterle, Qi Huang

**Affiliations:** Floral and Nursery Plants Research Unit, United States National Arboretum, Agricultural Research Service, United States Department of Agriculture, Beltsville, MD, United States

**Keywords:** *Ralstonia solanacearum*, cool-virulence, virulence, seedling screen, type III effector, RipS1

## Abstract

Members of the *Ralstonia solanacearum* species complex cause a variety of wilting diseases across a wide range of hosts by colonizing and blocking xylem vessels. Of great concern are race 3 biovar 2 strains of *R. solanacearum* capable of causing brown rot of potato at cool temperatures, which are select agents in the United States. To gain a better understanding of cool-virulence mechanisms, we generated libraries of transposon mutants in the cool-virulent *R. solanacearum* strain UW551 and screened 10,000 mutants using our seedling assay for significantly reduced virulence at 20°C. We found several mutants that exhibited reduced virulence at 28 and 20°C and also mutants that were only affected at the cooler temperature. One mutant of the latter chosen for further study had the transposon inserted in an intergenic region between a type III secretion system effector gene *ripS1* and a major facilitator superfamily (MFS) protein gene. Gene expression analysis showed that expression of *ripS1* was altered by the transposon insertion, but not the MFS protein gene. An independent mutant with this insertion upstream of *ripS1* was generated and used to confirm virulence and gene expression phenotypes. The effector, RipS1, has unknown function and is part of a family of effectors belonging to the largest known type III effectors. The functional connection between RipS1 and cool-virulence of *R. solanacearum* UW551 suggests that RipS1 (and/or its upstream promoter element) may serve as a potential target for development of cool-virulence-specific diagnostic tools to differentiate the highly regulated cool-virulent strains from non-cool-virulent strains of *R. solanacearum*. Our results provide important information for continued work toward a better understanding of cool-virulence of *R. solanacearum* and development of proper control strategies to combat this important plant pathogen.

## Introduction

Collectively, the *Ralstonia solanacearum* species complex (*Rssc*) – a group of related Gram-negative beta-proteobacteria – is pathogenic to many plant species, including several crops important economically and to food security across the globe (Hayward, 1991). The *Rssc* persists in soil and invades host plant roots, ultimately building to high-density populations in xylem vessels, which causes a reduction in water flow from roots to aerial tissues and frequently results in wilt symptoms (Lowe-Power et al., 2018). Although many strains in the *Rssc* are highly effective pathogens in their native tropical climates, some strains are of great economic concern in more temperate settings because of the ability to effectively infect, colonize, and cause disease symptoms at cool temperatures (<25°C), a phenotype referred to as cool-virulence (Janse et al., 1998; Van Elsas et al., 2000). Due to changing availability of techniques to characterize the members of the *Rssc*, classification has also changed over time. *Rssc* strains that exhibit the cool-virulence phenotype with potato as the host were initially designated as race 3 biovar 2 strains on the basis of host range and biochemical properties and subsequently designated as phylotype IIB sequevars 1 and 2 based on molecular characters from multiplex PCR (Fegan and Prior, 2005). Subsequently, nomenclature was updated and these strains belong to the “true” *R. solanacearum* species (Safni et al., 2014). Whereas genetic markers and diagnostic assays have been developed for detection and classification of *R. solanacearum* based on phylogeny, diagnostic tools based on markers directly connected to the cool-virulence phenotype are lacking and the specific underlying mechanisms enabling cool-virulence remain poorly understood. Recently, we identified a DNA region associated with the cool-virulence of *R. solanacearum* strain UW551 (Stulberg et al., 2018), and we also developed the first PCR assay targeting the cool-virulence associated region for specific detection and identification of race 3 biovar 2 strains of *R. solanacearum* (Stulberg et al., 2018).

Many virulence-associated traits have been identified in the *Rssc* and some of the most important include exopolysaccharide production, motility, and type III secretion (Meng, 2013). These traits enable the *Rssc* to migrate to susceptible hosts, attach to and invade roots, finally entering and colonizing xylem vessels (Álvarez et al., 2010). Exopolysaccharides produced by *Rssc* members provide a matrix facilitating and supporting bacterial growth, especially in host vascular tissues (Araud-Razou et al., 1998). *Rssc* strains exhibit both flagellar and twitching motilities; however, evidence suggests that twitching motility may play a greater role in overall virulence (Kang et al., 2002; Corral et al., 2020). Twitching motility at cool temperatures has been associated with cool-virulent *R. solanacearum* and may have direct or indirect effects on the cool-virulence phenotype (Bocsanczy et al., 2012; Stulberg et al., 2018).

As for many plant pathogens, the *Rssc* type III secretion system plays a critical role in virulence as it enables direct delivery of effector proteins from the bacterial cytoplasm into host cytoplasm (van Gijsegem et al., 1995). Type III effector proteins are involved in host defense suppression and manipulation of host physiological processes such as ubiquitin ligases and acetyltransferases to favor bacterial proliferation (Deslandes and Genin, 2014). The *Rssc* pan-genome includes a large arsenal of type III effectors, and efforts to confirm and characterize these effectors have identified certain effectors with critical functions for virulence, including some responsible for host-specificity (Poueymiro et al., 2009; Mukaihara et al., 2010). Some effectors are conserved and present in single copies in nearly all *Ralstonia*, such as RipU or the pectate lyase, and RipW (Sabbagh et al., 2019). Many *Rssc* type III effectors are grouped into families of similarly structured effectors with the genome of each *Rssc* strain carrying many members of the effector family. The RipS or SKWP effector family is characterized by carrying an array of 10–20 repeats of a semi-conserved 42 amino-acid motif that begins with the consensus SKWP, making them the largest known type III effectors (Mukaihara and Tamura, 2009). A member of this family, RipS1, has been identified several times in various efforts to describe the type III effector suite of the *Rssc* (Mukaihara and Tamura, 2009; Mukaihara et al., 2010; Peeters et al., 2013). As a result, RipS1, SKWP1, rip27, and hpx37 are all synonymous appellations that all refer to this single gene and corresponding locus tags are Rsc3401 and RRSL_04182 in strains GMI1000 and UW551, respectively. In a screen of several *Ralstonia pseudosolanacearum* type III effectors, it was determined that RipS1 can be induced to be secreted *in vitro*, and the occurrence of such secretion is dependent on the HpaB chaperone (Mukaihara et al., 2010). Additional work determined that RipS1 is not only secreted, but also is actually translocated into plant cells, in an HrpB dependent manner (Mukaihara and Tamura, 2009). Homologous effectors from the RipS family are present not only in *Ralstonia*, but also in other plant associated bacteria from *Xanthomonas*, *Rhizobium*, and even a few *Pseudomonads* (Teper et al., 2016). Deletion mutants of RipS family effectors, including *ripS1* in *R. pseudosolanacearum* and *xopAD* in *Xanthomonas citri* pv. citri have shown no virulence defect when this effector is lacking (Escalon et al., 2013; Lei et al., 2020). Indeed, the dispensability of these effectors is confirmed by the identification of natural isolates containing premature stop codons and insertion sequences likely resulting in loss of function (Escalon et al., 2013; Teper et al., 2016). An *R. pseudosolanacearum* strain lacking all RipS family effectors similarly had no observable virulence defect (Lei et al., 2020), and in plants inoculated with a 1:1 mix of wild-type and *ripS1* mutant bacteria, both strains were recovered at the same rate indicating no competitive disadvantage from the loss of this effector (Chen et al., 2018).

Many virulence-associated traits are expressed under control of complex regulatory networks, including quorum sensing, and critical transcription factors such as PhcA and HrpG (Vasse et al., 2000; Genin et al., 2005). Genes encoding effector proteins and structural components of the type III secretion system are expressed at high levels under *in planta* conditions (Coll and Valls, 2013). These genes are often co-regulated through a shared promoter element called a plant-inducible-promoter (PIP), characterized by a conserved sequence motif termed a PIP-box that has a consensus sequence of TTCGC – N_15_ – TTCGC (Cunnac et al., 2004). Presence of a PIP-box element in the promoter region of a gene is often an indication of important virulence function.

In this study, we implemented a high-throughput seedling virulence assay to screen for random transposon mutagenesis-generated *R. solanacearum* mutant strains for significantly reduced virulence under cool (20°C) but not warm (28°C) temperature conditions. Through our screen, we identified a transposon insertion mutant of interest that implicates the type III secretion effector gene *ripS1* in the cool-virulence phenotype. Expression of this type III effector gene is dysregulated in our transposon mutant, suggesting that appropriate expression of this effector is necessary for cool-virulence of *R. solanacearum* strain UW551.

## Materials and Methods

### Culture Conditions, Strains, and Plasmids

*Ralstonia solanacearum* strains from frozen stocks were routinely grown in casamino acid peptone glucose (CPG) culture medium (10 g L^−1^ peptone, 1 g L^−1^ yeast extract, 1 g L^−1^ casamino acids, and 5 g L^−1^ glucose) at 28°C and *Escherichia coli* strains were grown using LB culture medium (10 g L^−1^ tryptone, 5 g L^−1^ sodium chloride, and 5 g L^−1^ yeast extract) at 37°C, except where specified otherwise. The antibiotics kanamycin, ampicillin, and chloramphenicol were included in final concentrations of 30, 50, and 10 μg/ml, respectively, as appropriate. Supplementary Table S1 contains a list of strains and plasmids used in this study.

### Transposon Mutagenesis and Selection for Mutants of *Ralstonia solanacearum* Strain UW551

Random transposon mutants were obtained through conjugation of a mini-Tn5 suicide-donor plasmid from *E. coli* into the cool-virulent *R. solanacearum* strain UW551. UW551 cells were transformed by electroporation with the low-copy, broad-host-range plasmid pSIM7 (Datta et al., 2006) to provide a means of selection against donor *E. coli* following conjugation. Our *Nicotiana glutinosa* seedling screen was used to verify that there was no significant difference in virulence between wild-type UW551 and UW551 carrying plasmid pSIM7. For conjugations, the recipient UW551 cells carrying plasmid pSIM7 were prepared by growing cultures for 48 h to stationary phase at 28°C. Donor *E. coli*, S17-1 carrying the mini-Tn5 plasmid pUT-miniTn5-*luxCDABE* (Weitz et al., 2001) or pBAMD1-2 (Martínez-García et al., 2014), was grown to exponential phase, and mixed in a 1:1 ratio with the recipient *R. solanacearum* cells. The pBAMD1-2 was a gift from Victor de Lorenzo (Addgene plasmid #61564; RRID::Addgene61564).^1^ The cell mix was collected by centrifugation, washed twice with sterile water, and then resuspended in 1/20th volume of sterile water. Washed cells were dripped onto the surface of a CPG plate lacking any glucose or antibiotics, allowed to dry, and incubated at 28°C for 18 h. Cells grown on the CPG plate were resuspended in 15% glycerol and the transposon mutant library was stored at −80°C. When ready for use, selections were made by plating onto CPG containing chloramphenicol and kanamycin to select for *R. solanacearum* receiving the transposon. Dilutions were made so that single colonies grew with separation and were incubated at 28°C until colonies grew. Individual colonies were picked from selection plates and used directly as distinct UW551 mutant isolates for virulence screen using *N. glutinosa* seedlings as described previously (Schachterle and Huang, 2021). Briefly, *N. glutinosa* seeds were sown quickly and easily on top of individual soft water agar wells of a 96-well microtiter plate by pipetting out desired number of seeds in an aqueous suspension. They were inoculated on the same day by first touching a bacterial colony with an autoclaved toothpick and then stabbing the toothpick into the center of the water agar well (Schachterle and Huang, 2021).

### High-Throughput Virulence Screening of Transposon Mutants of *R. solanacearum* Strain UW551

Our recently developed *N. glutinosa* seedling screen (Schachterle and Huang, 2021) was used for high-throughput screening of *R. solanacearum* UW551 transposon mutants for significantly reduced virulence at 20°C. Each mutant colony was inoculated into an individual well containing *N. glutinosa* seeds as described above. Wild-type UW551 and sterile water were used for inoculation as positive and negative controls, respectively. The inoculated 96-well plates were incubated at 20°C for 18–21 days before evaluation of symptoms. Mutants in wells causing visibly reduced disease symptoms compared to the wild-type UW551 at 20°C were selected for additional testing. This was done by re-isolation of the selected mutant isolate from its water agar well by streaking from the water agar well onto solid CPG media, providing colonies to repeat the virulence assay using seedlings in three replicate wells at 20 and at 28°C to confirm its reduced virulence was specific only to the cool but not to the warm temperature.

### Virulence Assay Using Tomato Seedlings

Virulence of *R. solanacearum* on tomato seedlings was tested using two approaches. In one approach, seedlings were not wounded, and seeds were sown in soft water agar and inoculated with the selected mutant isolate on the day of sowing, incubated at the indicated temperature and monitored for growth and symptom development. This approach mirrors what was done in the *N. glutinosa* seedling screen, except with a different host, and was used to confirm the temperature-specific virulence phenotype of the mutant isolate in *N. glutinosa*. This approach was also used to extract RNA from inoculated tomato seedlings at different temperatures for gene expression analysis described below. In the other approach (Khokhani et al., 2018), young tomato seedlings were grown in soil at 28°C. After wounding by removal of a leaf, and inoculation with 2 μl of a 10^5^ bacterial cells/ml suspension on the petiole of the removed leaf to directly introduce the selected *R. solanacearum* strain to the xylem, the inoculated tomato seedlings were incubated at the indicated temperature and monitored daily for disease symptoms. This approach was used to determine whether delivering the mutant isolate directly into the plant has any effect on the temperature-specific virulence of the mutant isolate.

### Phenotypic Trait Assays

Swimming motility was assayed as described (Corral et al., 2020). Briefly, overnight cultures of *R. solanacearum* were washed with sterile water and adjusted to an OD_600_ of 0.1, and 3 μl of this bacterial cell suspension were stab inoculated into the center of the soft-agar medium plate (10 g/L peptone, 2.5 g/L agar). The plate was then incubated at the indicated temperature for 24 h and imaged. The halo of cells swimming from the point of inoculation was quantified using ImageJ analysis software (Abràmoff et al., 2004). Susceptibility to exogenous hydrogen peroxide was conducted as described previously (Schachterle et al., 2019), with the modification that CPG medium was used instead of LB. Briefly, overnight cultures of *R. solanacearum* were washed with sterile water and diluted to an OD_600_ of 0.1. Then, 50 μl of the cell suspension was spread on a CPG plate. An 8 mm sterile filter paper disk was placed in the center of the plate on top of the CPG plate with cells and 10 μl of 1 M hydrogen peroxide was added to the filter paper, and plates were incubated at 20 or 28°C, as indicated. Plates were imaged after 48 h, and the zone of growth inhibition around the filter paper disk was quantified using ImageJ (Abràmoff et al., 2004). Twitching motility was assessed as described (Bocsanczy et al., 2012). Briefly, cells were diluted to grow individual colonies and grown on CPG medium at either 20°C for 48 h or 28°C for 24 h before analysis and imaging by microscopy. Each assay was repeated three times, each time with at least three replicates. For twitching motility, images shown are representative.

### Transposon Insertion Site Sequencing and Identification

Transposon insertion locations were determined using arbitrary PCR amplification followed by Sanger sequencing. Arbitrary PCR was conducted in two amplification reactions as described previously (Miller-Williams et al., 2006; Mendez et al., 2018). In the first round of PCR, a primer specific to one side of the transposon (MCS-Arb Ext; Supplementary Table S2, or TDKm6; Mendez et al., 2018) is used with a degenerate primer (DGEN1; Miller-Williams et al., 2006) under a low annealing temperature to amplify the edge of the transposon. In the second round, a nested primer specific to the transposon edge MCS-Arb Int or TDKm-Int (Supplementary Table S2) is used with a primer specific to the degenerate primer (DGEN2; Miller-Williams et al., 2006) to provide amplification specificity to the transposon edge. All oligonucleotide sequences are included in Supplementary Table S2. Purified PCR products were submitted to Eurofins Genomics (Louisville, KY) for sequencing using the transposon-specific nested primer (MCS-Arb Int or TDKm-Int). Positions of flanking sequences were determined using blastn from the BLAST+ suite (Camacho et al., 2009) with the UW551 genome (Hayes et al., 2017) as database.

### RNA Isolations

For RNA isolations from culture medium, *R. solanacearum* strains were grown overnight in CPG medium, washed, and resuspended to an OD_600_ of 1. For trials in CPG medium, 500 μl cells were resuspended in sterile water and diluted 100-fold into CPG and incubated at indicated temperature with 200 rpm agitation. For trials in minimal medium, 2 ml cells were resuspended in minimal medium and diluted 4-fold into minimal medium and incubated at the indicated temperature with 200 rpm agitation. After 24 h of growth, cells were collected by centrifugation and RNA was isolated using an approach as described (Rivas et al., 2001). For RNA isolations from tomato seedlings, seeds were sown in soft water agar (0.2% w/v) and the water agar was stab-inoculated on the same day as described above. Seven days post-sowing/inoculation, seedlings were collected and total RNA was isolated using the E.Z.N.A. Plant RNA kit (Omega Bio-tek, Norcross, GA, United States) according to manufacturer’s instructions. RNA quality and quantity were assessed using agarose gel electrophoresis and spectrophotometrically *via* NanoDrop (Thermo Scientific, Waltham, MA, United States).

### qPCR and Gene Expression Analysis

RNA was reverse transcribed to cDNA using the High Capacity cDNA Reverse Transcription kit (Applied Biosystems, Foster City, CA, United States) following manufacturer’s protocol with random hexamers. Quantitative real-time PCR (qRT-PCR) was conducted using cDNA as template for reactions with iQ SYBR Green Supermix (Bio-Rad Laboratories, Hercules, CA, United States) and run on a C1000 Thermal Cycler (Bio-Rad Laboratories, Hercules, CA, United States). Three biological replicate cultures were used for each strain-temperature-medium combination for RNA isolation and cDNA synthesis. Each cDNA sample was in turn tested with three technical replicates per primer set in qPCR reactions. Gene expression was calculated using the ddCt method (Livak and Schmittgen, 2001) with an established 16S rRNA primer set as endogenous control (Addy et al., 2012).

### Generation and Testing of Independent Mutant UW551-TnR1-ck

To confirm that the observed phenotypes in the transposon mutant UW551-TnR1 were caused by insertion of the transposon at the determined insertion site, not spontaneous mutations at other genomic positions, an independent mutant containing the exact transposon insertion at the same genomic site in UW551 was generated by site-directed double homologous recombination. Briefly, the transposon and flanking genomic DNA were amplified from UW551-TnR1 genomic DNA by high-fidelity PCR with Phusion polymerase (New England Biolabs, Ipswich, MA, United States) using the *RRSL04180* forward primer and the *ripS1* qPCR reverse primer. The resulting PCR fragment contained the transposon insertion site – which carries kanamycin resistance – with 688 nt and 1,391 nt of DNA flanking the transposon on the side of *ripS1* or *RRSL_04180*, respectively, allowing for homologous recombination to independently introduce the mutation to wild-type strain UW551. To prepare electrocompetent cells, overnight cultures of strain UW551 grown in CPG medium were iced, washed five times with ice-cold sterile water, and resuspended in a 0.1X volume, concentrating the cells 10-fold from the original culture density. PCR products were purified using the QIAquick PCR purification kit according to manufacturer’s instructions (Qiagen, Germantown, MD, United States), and 10 μg of PCR product was added to 50 μl of competent UW551 in a pre-chilled electroporation cuvette (2 mm gap width). Electroporation was conducted using a MicroPulser (BioRad, Hercules, CA, United States) on setting EC2. Following electroporation, 1 ml of CPG medium was added to the cuvette and recovery of cells was conducted at 28°C for 4 h. Following recovery, cells were plated onto CPG plates containing kanamycin to select for recombinant mutants and incubated at 28°C until colonies grew. A colony that was confirmed by PCR and Sanger sequencing to contain the transposon insertion at the same flanking sequences as UW551-TnR1 was selected and designated UW551-TnR1-ck. Virulence testing of this strain was conducted using seedlings of *N. glutinosa* and tomato grown in soft water agar as described above. RNA isolation, cDNA synthesis, and qPCR for gene expression analysis of UW551-TnR1-ck was conducted as described above.

## Results

### Generation of Transposon Mutants of *R. solanacearum* UW551 and Screen for Mutants With Reduced Virulence

**Figure 1:**
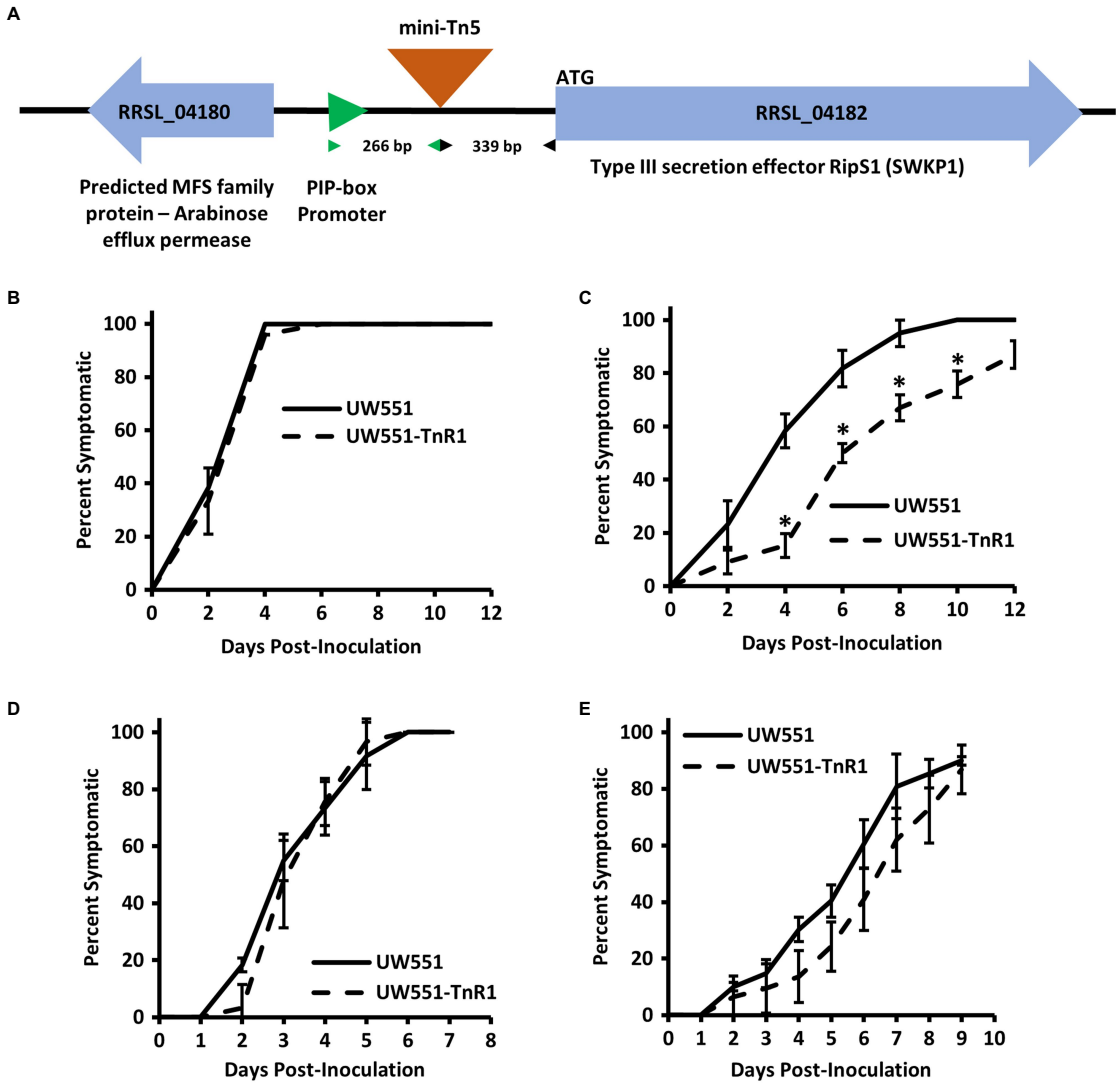
Intergenic transposon insertion in mutant strain UW551-TnR1 reduces virulence at 20°C in tomato seedlings, but not when the mutant was introduced directly to the xylem. Schematic diagram showing the insertion site of the mini-Tn5 transposon in UW551-TnR1 between the major facilitator superfamily (MFS) protein gene *RRSL_04180* and *ripS1* in parental UW551, as well as the distance between the transposon insertion and associated plant-inducible-promoter (PIP) element and *ripS1*, respectively **(A)**. Confirmation that mutant UW551-TnR1 recovered from *Nicotiana glutinosa* seedling screen exhibits full virulence at 28°C **(B)** and reduced virulence at 20°C **(C)** in tomato seedlings. No significant difference in virulence was observed between wild-type UW551 and mutant UW551-TnR1 at individual time points when each bacterium was inoculated directly to tomato xylem through a wounded petiole regardless of whether grown at 28 **(D)** or 20°C **(E)**. Experiments were repeated four times, each time with at least five seedlings across two wells of a 12-well plate **(B,C)** or five seedlings grown in soil **(D,E)**. Each individual seedling was assessed for any symptoms, including wilt, water soaking of leaves, collapse of stem, or brown discolored lesions on stem, and those showing symptoms were calculated as percent of all seedlings in that treatment group. Values show average of all four experiments and error bars represent SE. Asterisks denote statistically significant difference (*p* < 0.05) from wild-type strain UW551 at the indicated timepoint by Student’s *t*-test.

We generated libraries of random mini-Tn5 transposon insertion mutants by conjugating transposon donor plasmid pUT-miniTn5-*luxCDABE* or pBAMD1-2 into the cool virulent *R. solanacearum* strain UW551 (Supplementary Table S1). We then employed our recently developed *N. glutinosa* seedling virulence assay (Schachterle and Huang, 2021) to screen 10,000 isolates from our mutant libraries. The screen was done at 20°C first and 302 isolates exhibiting reduced virulence were chosen for further testing. These 302 isolates were tested with additional replicates at both 20 and 28°C. Of these, 16 had reduced virulence at both temperatures tested and 10 isolates had reduced virulence only at 20°C, but not 28°C. Of the 26 mutants of interest collected from our screen, arbitrary PCR followed by Sanger sequencing was successful for 20 mutants, indicating a single transposon insertion. Of particular interest, we obtained three mutant isolates that all had an identical transposon insertion, potentially arising from a single transposon insertion event followed by cell division before selection pressure was imposed. This transposon insertion was located on the UW551 chromosome at nucleotide 3,408,846 (assembly GCA_ 002251655.1) in an intergenic region between a major facilitator superfamily (MFS) protein gene (*RRSL_04180* in assembly GCA_000167955.1; *B7R79_15985* in assembly GCA_002251655.1) and a type III secretion system effector gene, *ripS1* (*RRSL_04182* in assembly GCA_000167955.1; *B7R79_15995* in assembly GCA_002251655.1) 339 bases upstream of the *ripS1* start codon (Figure 1A). We designated this mutant strain as UW551-TnR1 and chose this mutant for further study because of its proximity to a type III effector.

To confirm the temperature dependent effects on virulence of the transposon insertion in UW551-TnR1, we assessed virulence of the mutant strain using seedlings of tomato, a commonly and widely used host of *R. solanacearum*, grown on water agar at 20 or 28°C. Similar to *N. glutinosa* seedling results, we observed that in tomato seedlings grown at 28°C, the mutant displayed full virulence compared to wild-type UW551, but at 20°C, seedlings inoculated with the mutant showed delayed appearance of disease symptoms such as stem discoloration and lesions, water soaking, and wilt compared to the wild-type strain (Figures 1B,C).

### Direct Introduction of Mutant UW551-TnR1 Into Tomato Xylem Overcomes Reduced Cool Virulence Phenotype in the Mutant

Because infection and symptom development in our seedling assay requires bacterial movement to the seedling root and invasion of growing plant tissues, it is possible that the cool virulence defect observed in UW551-TnR1 is due to some defect in an early-stage infection process. To determine whether the UW551-TnR1 cool-virulence defect occurs before or after bacterial entry into the host plant, we introduced the bacterial inoculum directly to the xylem of tomato plants *via* a wounded petiole. We observed no significant differences in virulence between the wild-type strain UW551 and the mutant strain UW551-TnR1 at individual timepoints regardless of whether the virulence assay was done at 20 or 28°C (Figures 1D,E).

We further assessed the UW551-TnR1 mutants for various traits potentially involved in cool-virulence in early stages of infection. We found that the mutant strain was unaltered compared to the wild-type strain for flagellar motility, twitching motility, or susceptibility to exogenous hydrogen peroxide, regardless of whether grown at 20 or 28°C (Supplementary Figure S1).

### Transposon Insertion in *R. solanacearum* Strain UW551 Alters Expression of *ripS1* but Not *RRSL_04180*

**Figure 2:**
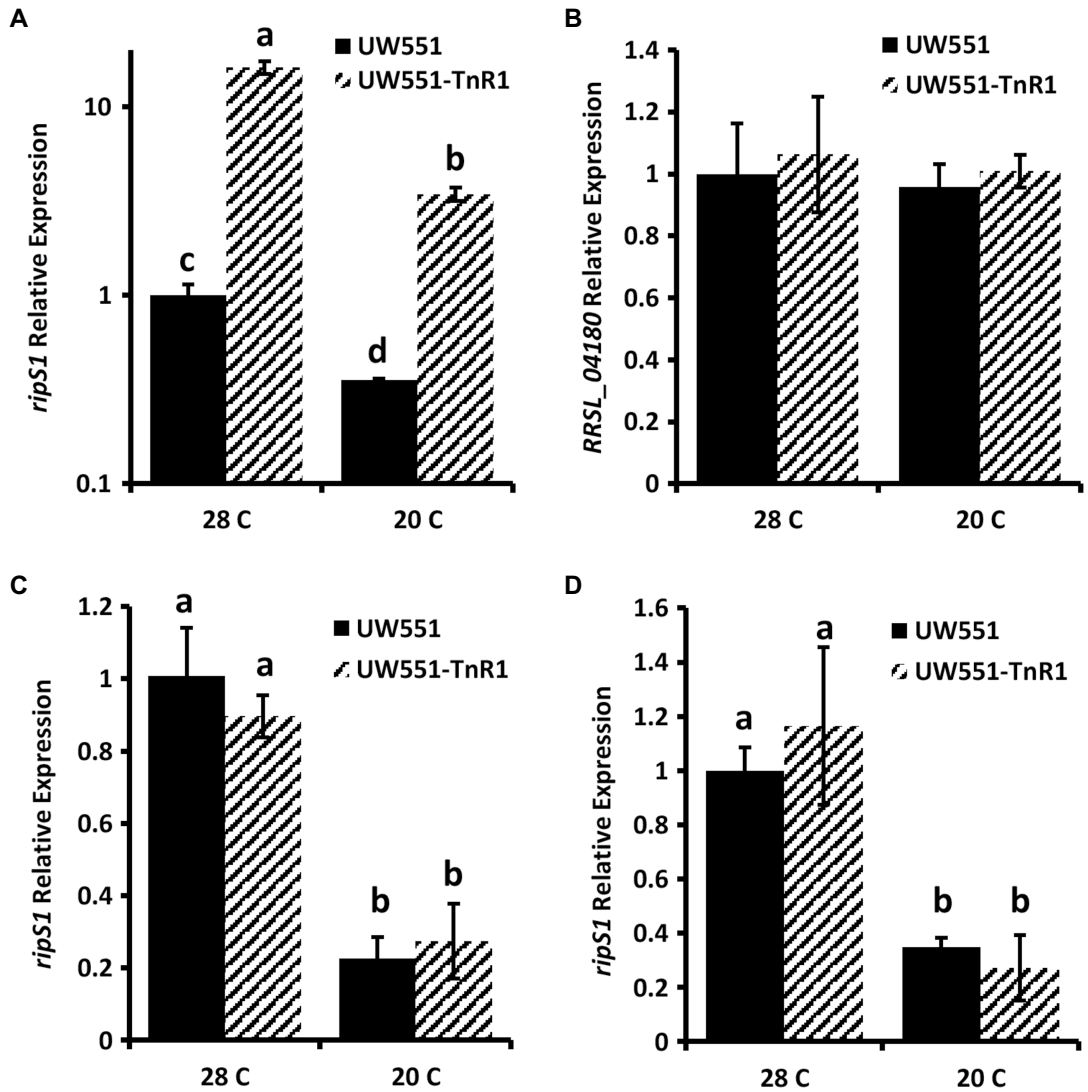
Relative mRNA abundance in wild-type UW551 or transposon mutant UW551-TnR1 of *ripS1* **(A,C,D)** or *RRSL_04180* **(B)**. cDNA from RNA isolated from bacterial cells grown for 24 h in CPG medium **(A,B)**, in minimal medium **(C)** or using RNA from infected tomato seedlings **(D)** grown at 28 or 20°C served as template for quantitative real-time PCR (qRT-PCR) reactions. Three independent samples were collected for each strain-growth combination, and each sample was tested in triplicate. Values represent average of all samples and error bars represent SD. Samples with different letters are significantly different (*p* < 0.05, Student’s *t*-test, all combinations).

To determine any effect of the transposon insertion in the mutant strain UW551-TnR1 on expression of the neighboring genes, we assessed their expression by qRT-PCR. We found that in cells grown in CPG medium at 28°C, expression of the *ripS1* effector gene was increased by about 10-fold in the UW551-TnR1 mutant compared to the wild-type strain UW551 (Figure 2A). When compared to cells grown in CPG medium at 20°C, the decrease in temperature resulted in decreased abundance or *ripS1* mRNA by a factor of 2–3-fold in both the UW551-TnR1 mutant and wild-type UW551 cells, with the UW551-TnR1 still about 10-fold higher than UW551 (Figure 2A). Expression of the MFS gene (*RRSL_04180*) on the other side of the transposon insertion was unaffected between wild-type UW551 and mutant UW551-TnR1 (Figure 2B). To ensure that no systematic bias was introduced in our sample treatment methods, we tested two other type III effector genes, one located on the *R. solanacearum* chromosome and one located on the megaplasmid, *ripW*, and *ripU*, respectively. Neither *ripW* nor *ripU* showed temperature dependent expression, and their expression was not significantly altered by the transposon insertion (Supplementary Figure S2).

**Figure 3:**
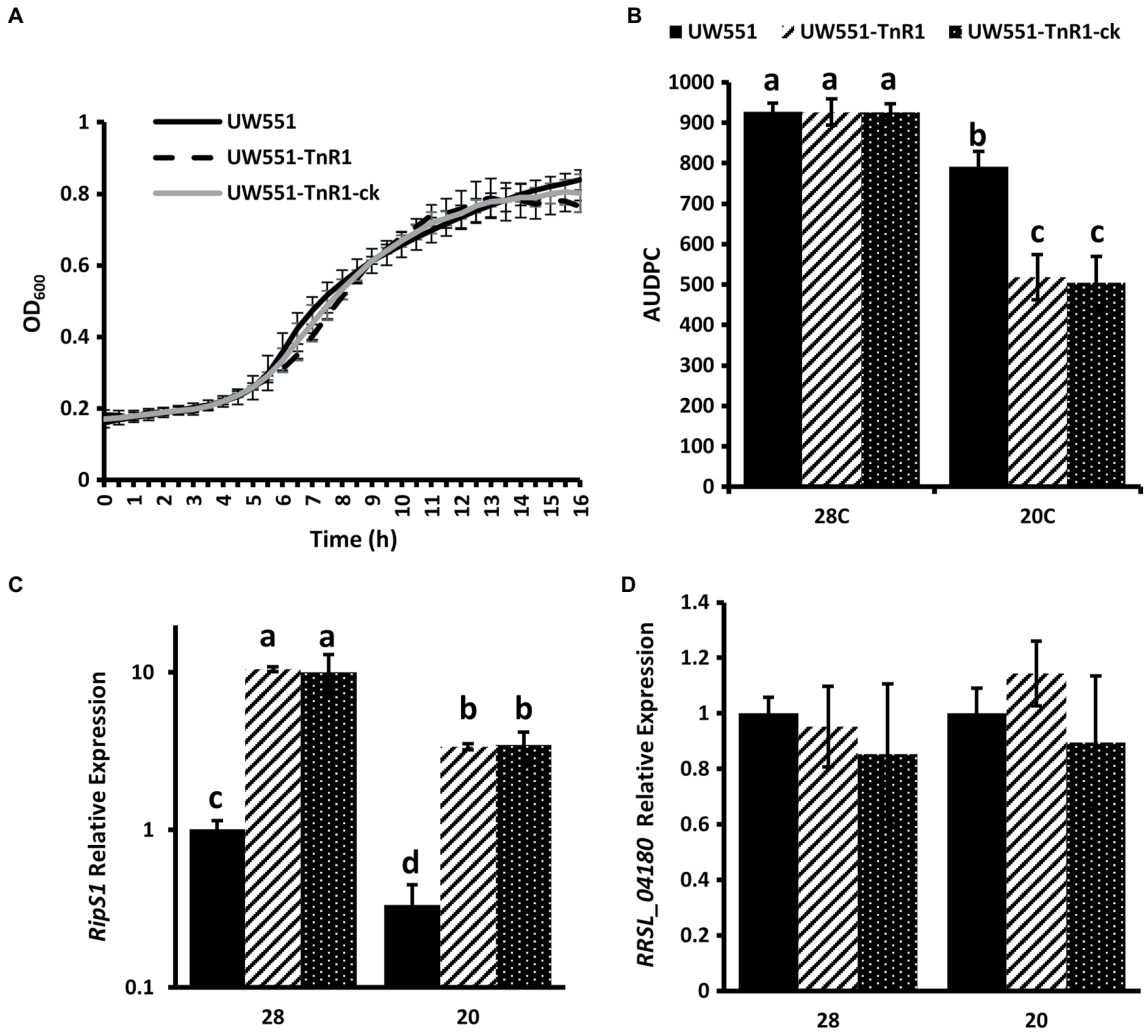
Independent mutant UW551-TnR1-ck confirms phenotypes of random transposon mutant UW551-TnR1. No significant differences observed in growth in CPG medium **(A)**. Area under the disease progress curve (AUDPC) for virulence assays in tomato seedling assays shows no difference between transposon mutant UW551-TnR1 and independent mutant UW551-TnR1-ck, as compared to the wild-type strain UW551 **(B)**. Similar relative mRNA abundance of *ripS1* **(C)** or *RRSL_04180* **(D)** in transposon mutant UW551-TnR1 and independent mutant UW551-TnR1-ck, as compared to the wild-type strain UW551. cDNA from RNA isolated from bacterial cells grown for 24 h in CPG medium at the indicated temperature served as template for qRT-PCR reactions. Three independent samples were collected for each strain-growth combination, and each sample was tested in triplicate. Values represent average of all samples and error bars represent SD. Samples with different letters are significantly different (*p* < 0.05, Student’s *t*-test, all combinations).

To confirm that the phenotypes of the UW551-TnR1 mutant were due to single transposon insertion and not due to mutations at other locations in the genome, we generated an independent mutant containing the same transposon insertion at nucleotide 3,408,846 in UW551 using homologous recombination methods. This mutant, designated UW551-TnR1-ck did not differ significantly in growth from UW551-TnR1, and neither of these mutant strains differed significantly from wild-type UW551 in growth (Figure 3A). The virulence patterns observed in the independent mutant UW551-TnR1-ck did not differ significantly from those of UW551-TnR1 (Figure 3B), and similar gene expression patterns for *ripS1* and *RRSL_04180* were also observed in UW551-TnR1-ck as in UW551-TnR1 (Figures 3C,D).

**Figure 4:**
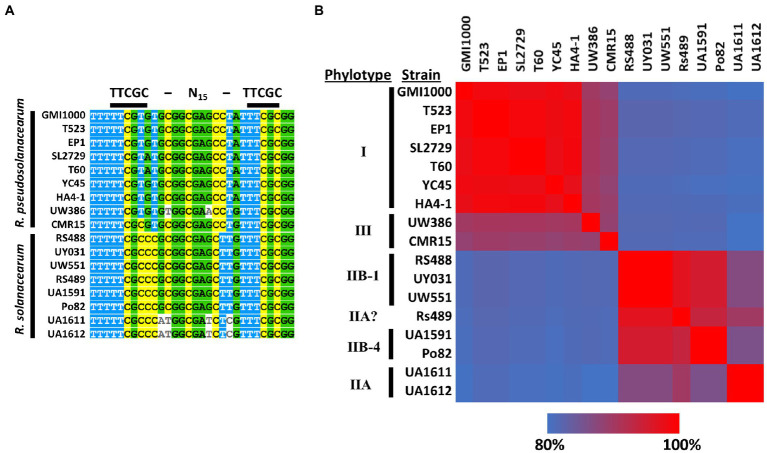
Multiple sequence alignment of PIP-box located about 600 bases upstream of *RipS1* in various *Ralstonia solanacearum* species complex (Rssc) strains shows that phylotype II strains harbor a perfect PIP-box consensus match at this site **(A)**. Heatmap showing percent identity for the intergenic region between *RRSL_04180* and *ripS1* in various *R. solanacearum* species complex strains indicates that phylotype II strains clearly separate from phylotypes I and III in this region and IIB-1 strains can be separated from all other strains **(B)**. Multiple sequence alignments were generated and percent identity calculated using MAFFT.

A PIP-box upstream of *ripS1* was predicted in strain GMI1000 (Mukaihara and Tamura, 2009), suggesting that *ripS1* is likely co-regulated with type III secretion system genes. We generated a multiple sequence alignment of the *ripS1* PIP-box from several *R. solanacearum* and *R. pseudosolanacearum* strains and found that it is conserved and that the sequence for *R. solanacearum* strains are a better PIP consensus match than for *R. pseudosolanacearum* strains (Figure 4A). We also performed a multiple sequence alignment of the entire intergenic region between RRSL_04180 and *ripS1* from a variety of *Ralstonia* strains and found clear separation between *R. solanacearum* and *R. pseudosolanacearum* (Figure 4B). *Ralstonia solanacearum* strain clustering further separated phylotype IIB sequevar 1 from sequevar 4 and both were separated from phylotype IIA strains (Figure 4B). Strain Rs489, which has previously proven difficult to classify (Stulberg et al., 2018), grouped near the phylotype IIB sequevar 1 strains, but does not contain identical sequence (Figure 4B). Grouping based on multiple sequence alignments of the amino acid sequence of effector RipS1 shows similar patterns but with phylotype IIB sequevar 4 grouping closer to the phylotype IIB sequevar 1 strains than strain Rs489 (Supplementary Figure S3).

### Transposon Insertion Effect Lost Under Type III Secretion Inducing Conditions

To test if the transposon insertion in UW551-TnR1 altered *ripS1* expression under conditions in which the PIP-box is predicted to be active, we grew cells for RNA extraction and qPCR analysis using a minimal medium in which *R. solanacearum* has been shown to express type III secretion system genes at high levels (Arlat et al., 1992). We found no significant difference in *ripS1* expression between wild-type and mutant cells grown at the same temperature (Figure 2C). However, the temperature dependent difference in expression was still manifest, since for both strains, expression of *ripS1* was higher at 28°C, compared to at 20°C (Figure 2C). We further tested *ripS1* expression during infection of tomato seedlings to determine whether host-specific factors would have a different effect. In these samples, we again observed temperature dependent differences between growth at 20 and 28°C but like in minimal medium there was no difference between wild-type and mutant infected samples under the same temperature (Figure 2D).

## Discussion

Using a newly developed high-throughput virulence screening method (Schachterle and Huang, 2021) to screen 10,000 mutants of *R. solanacearum* strain UW551 in *N. glutinosa* grown in water agar wells of 96-well plates led to the discovery of 26 mutants with reduced virulence, suggesting the effectiveness of the screening method. One of the mutants, UW551-TnR1, was chosen for further study and displayed significantly reduced virulence under cool temperatures but maintained full virulence under warm temperature conditions in the screen assay, and such virulence phenotype was confirmed by subsequent tomato seedling assays.

The mutant UW551-TnR1 contains a transposon insertion between the gene encoding the type III effector RipS1 and its predicted PIP promoter. This insertion resulted in increased expression of *ripS1* when grown in rich medium, but no difference under type III secretion inducing conditions. This suggests that the transposon insertion is causing a constitutive activation of *ripS1*. Our data indicate that this dysregulation, or inability of *R. solanacearum* to shut down expression of this gene, is leading to a reduction in virulence at 20°C, but that these bacteria are fully virulent at 28°C. Significant reduction in virulence was not observed when a high dose of bacterial cells were directly introduced to tomato xylem, suggesting the role of RipS1 during early infection processes. Indeed, recent work provides evidence that certain RipS effectors, including RipS1 may play more important virulence roles than other members of this family during infection of eggplant (Chen et al., 2021). However, we observed no change in the virulence traits of swimming or twitching motility, and no change in susceptibility to exogenous oxidative stress from hydrogen peroxide in mutant UW551-TnR1 compared to wild-type UW551. While other early-stage infection virulence traits may play a role, such as ability to spread between xylem vessels, our data suggest that the specific function of RipS1 as an effector may be responsible for the reduction in cool virulence and not pleiotropic effects on other virulence traits tested in this study. Our further work to confirm virulence and gene expression phenotypes using the independently generated strain UW551-TnR1-ck provides additional evidence that this effect appears to be through RipS1, whether directly or indirectly, and not any other spontaneous mutations that may have arisen during transposon mutagenesis.

Because several deletion mutants lacking RipS family effectors have no virulence defect (Lei et al., 2020), our data suggest that the significantly reduced virulence, we observed in mutant UW551-TnR1 is not because of any loss of function of RipS1, but is more likely due to an inappropriate expression at some critical stage of cool-virulence or in some subpopulation of cells. However, it is noteworthy that the studies conducted with *ripS* deletion mutants were in non-cool virulent *R. pseudosolanacearum* and were not tested at cool temperature, and functions of *ripS* at different temperature conditions in different *Ralstonia* species may be different.

Our results indicate that *ripS1* is expressed at lower levels at 20°C compared to 28°C. A transcriptomic study of strain UW551 grown in CPG medium also found a 2.33-fold decrease in *ripS1* expression at 20°C compared to 28°C (Meng et al., 2015). However, other transcriptomic and genomic studies did not associate *ripS1* with differential expression based on temperature or *in planta* growth or differential presence between strains (Jacobs et al., 2012; Bocsanczy et al., 2017). Similarly, RipS1 was not detected in a proteomic study of *R. solanacearum* at different temperatures (Bocsanczy et al., 2014), but this is not surprising given its nature as a secreted protein, that other type III effectors were not detected, and the difficulty of consistent detection of low-abundance proteins in shotgun proteomic analyses. The difficulties of these other genome-scale approach to converge on factors associated with the cool-virulence magnifies an advantage of our use of a forward genetic screen to specifically test for the cool-virulence phenotype.

A PIP-box was predicted 683 bases upstream from the *ripS1* start codon in strain GMI1000 (Mukaihara and Tamura, 2009), and we found that PIP-box is conserved in the promoter region of *ripS1* in *R. solanacearum* and *R. pseudosolanacearum*, with *R. solanacearum* strains perfectly matching the PIP consensus sequence (Figure 4A). Although this promoter, and associated transcriptional start site suggested by the PIP-box have not been experimentally validated, this would represent a very long 5´UTR that could be subject to many types of regulation, including post-transcriptional regulation (Oliva et al., 2015). Indeed, it is in this region that the transposon inserted in UW551-TnR1. The increased mRNA abundance due to uncoupling of the PIP-box from *ripS1* suggests that there are likely binding sites for other transcription factors or other regulatory elements in the *ripS1* promoter and 5´UTR region. Our data support this because regardless of the presence of the transposon insertion or cell growth conditions, we observed temperature dependence in *ripS1* mRNA abundance but no temperature dependence in other type III effectors *ripW* and *ripU*. Consistent with other findings, our results do not point to the well-characterized type III secretion regulatory systems as temperature dependent, but suggest another mechanism exists regulating temperature dependence. Additional work is needed to uncover the mechanism of temperature dependence in *ripS1* expression in cool-virulent strains of *R. solanacearum*.

Although the function of RipS family effectors remains unknown, a recent study predicted RipS1 and several other *R. solanacearum* type III effectors to carry nuclear localization signals (NLSs) that would enable them to localize to the plant host nucleus following translocation (Jeon et al., 2020). RipS1 and three other members of the RipS effector family were predicted to carry one or more NLSs. Although shown for other effectors with predicted NLSs, functionality was not determined for any members of the RipS effector family. Previous work has suggested that the cool-virulence phenotype of some *R. solanacearum* is dependent on interaction with temperature dependent host-derived factors (Milling et al., 2009). The significantly reduced virulence due to increased expression of *ripS1* at cool temperatures could fit this hypothesis, especially when no reduction in virulence was observed, while this effector was also overexpressed at warm temperature. Although not a well understood phenomenon, similar observations have been made in which overexpression of specific type III effectors *in planta* reduces pathogen fitness (Macho et al., 2012). However, we found no difference in *ripS1* mRNA abundance between UW551 and UW551-TnR1 under *hrp*-inducing conditions, such as in minimal medium or in tomato seedlings, regardless of temperature. Because of the possibility that *ripS1* expression changes over time and in different niche environments presented by the host, expression differences between UW551 and UW551-TnR1 may exist that were not observed due to our limited testing, which occurred at 7 days post-inoculation and after symptom appearance. Thus, unobserved differences in expression may still exist. Nonetheless, our data suggest that additional complexities must be involved to explain these apparently contradictory results. Several possibilities exist that could cause such a contradiction. These include post-transcriptional or post-translational regulations altering RipS1 activity without affecting mRNA abundance. Another possibility is localized expression of *ripS1* by a subpopulation of cells in different *in planta* environments, or only during specific stages of disease development such as during attachment, invasion, colonization, or systemic spread. Furthermore, epistatic interactions with additional genes could result in behaviors that are inexplicable when the expression of only *ripS1* is considered. Indeed, each of these potential complexities has been observed in plant pathogenic bacteria (Cui et al., 2018; Yuan et al., 2019), and support an abundance of evidence that indicates that cool-virulence is complex and likely controlled by a suite of genes, rather than a single actor.

Deletion of a previously identified genomic region that resulted in reduced cool-virulence also resulted in cool temperature reduction in twitching motility (Stulberg et al., 2018). Although associations between cool-virulent *R. solanacearum* and twitching motility have been established (Bocsanczy et al., 2012; Stulberg et al., 2018), our transposon mutant UW551-TnR1 did not exhibit any qualitative reduction in twitching motility at cool or warm temperatures indicating that likely multiple virulence factors contribute to the cool-irulence phenotype in *R. solanacearum*.

Our data implicate the type III effector RipS1 as playing an important role in cool-virulence of *R. solanacearum*. Additional work is needed to determine whether RipS1 has a direct role such as interacting with a specific host factor or protein at cool temperatures, or if it only has an indirect role in cool-virulence, such as overexpression of *ripS1* in the UW551-TnR1 mutant prevents efficient secretion of another type III effector that in turn has a direct role. Multiple sequence alignments indicate that the RipS1 protein and its associated promoter region can separate cool-virulent *R. solanacearum* (phylotype IIB sequevar 1) strains from others and may prove useful for development of diagnostic tools specific to cool-virulent *R. solanacearum*. Better understanding of the mechanisms underlying the cool-virulence phenotype will facilitate accurate definition, detection, and control of the select agent strains of *R. solanacearum* to safeguard United States Agriculture.

## Data Availability Statement

The original contributions presented in the study are included in the article/Supplementary Material, further inquiries can be directed to the corresponding author.

## Author Contributions

JS and QH conceived and designed the study, analyzed the data, and wrote the manuscript. JS conducted the experiments. All authors contributed to the article and approved the submitted version.

### Conflict of Interest

The authors declare that the research was conducted in the absence of any commercial or financial relationships that could be construed as a potential conflict of interest.

## Footnotes

^1^http://n2t.net/addgene:61564
