# Supplementary material for: Implication of the Type III Effector RipS1 in the Cool-Virulence of Ralstonia solanacearum Strain UW551
Source: Front Plant Sci. 2021 Jul 22;12:705717. doi: 10.3389/fpls.2021.705717 (PMC8339923; doi:10.3389/fpls.2021.705717)
Supplement: Supplementary file 1 [file Data_Sheet_1.PDF]

Supplemental Materials for

Implication of the type III effector RipS1 in the cool-virulence of *Ralstonia solanacearum* strain UW551

Jeffrey K. Schachterle & Qi Huang

<u>Contents</u> <u>page</u>

Table S1	2
Table S2	3
Figure S1	4
Figure S2	5
Figure S3	6

Table S1: Bacterial Strains and plasmids used in this study

Species/Strain	Relevant Characteristics	Source/Reference
Ralstonia solanacearum		
UW551	phy IIB seq 1	C. Allen, USA (Gabriel et al. 2006)
UW551-pSIM7	UW551 containing pSIM7; Cm^R	This Work
UW551-TnR1	UW551 mutant with mini Tn5 transposon inserted upstream of *ripS1*	This Work
UW551-TnR1-ck	Independently generated mutant of UW551 containing the mini Tn5 transposon insertion upstream of *ripS1*	This Work
Escherichia coli		
S17-1	pBAMD1-2; Km^R, Amp^R; suicide vector for transposon mutagenesis	Addgene (Martínez-García et al. 2014)
S17-1	pUT-miniTn5-*luxCDABE*; Km^R, Amp^R; suicide vector for transposon mutagenesis	Hedda J. Weitz, UK (Weitz et al. 2001)
DH5α	pSIM7; Cm^R; pBBR1 origin of replication, used for counter-selection in transposon mutagenesis	Donald L. Court, USA (Datta et al. 2006)

Table S2: Oligonucleotides used in this study

Oligo Name	Sequence	Purpose of Oligonucleotide	Source/Reference
DGEN1	GGCCACGCGTCGACTAGTCAGNNNN-NNNNNNACGCC	1st round of arbitrary PCR, degenerate	(Miller-Williams et al. 2006)
DGEN2	GGCCACGCGTCGACTAGTCAG	2nd round of arbitrary PCR, after DGEN1	(Miller-Williams et al. 2006)
MCS-Arb Ext	GGTACCGAGCTCGAATTCGCG	1st round of arbitrary PCR from left side of transposon	This Work
MCS-Arb Int	CCTAGGCGGCCTGAGACACAAAG	2nd round of arbitrary PCR and sequencing from left side of transposon	This Work
TDKm6	AGTTTCATTTGATGCTCGATG	1st round of arbitrary PCR from right side of transposon	(Mendez et al. 2018)
TDKm-Int	CCTTGTCTAATTAATTGCGGACCC	2nd round of arbitrary PCR and sequencing from right side of transposon	This Work
ripS1 qPCR F	AGCGCTGAACTACCCTGAGC	qPCR testing of *ripS1* expression	This Work
ripS1 qPCR R	CTCATGCGCCAGAGTTCG		This Work
RRSL_04180	ATGGACTTCATCGTGATGATGC	qPCR testing of *RRSL_04180* expression	This Work
RRSL_04180	CACGAATACGCCGACACG		This Work
ripU qPCR F	CGTGGTGTTGAAGGAGGAAT	qPCR testing of *ripU* expression	This Work
ripU qPCR R	ATGCACATAAGCCCCTGAAC		This Work
ripW qPCR F	CGAGTGACCATTTCCATGTG	qPCR testing of *ripW* expression	This Work
ripW qPCR F	GACCTGCTGGAGCAGTTGTA		This Work
16S qPCR F	CTAGAGTGTGTCAGAGGGAGGTAGA	qPCR testing, endogenous control	(Addy et al. 2012)
16S qPCR R	ATGTCAAGGGTAGGTAAGGTTTTTC		(Addy et al. 2012)

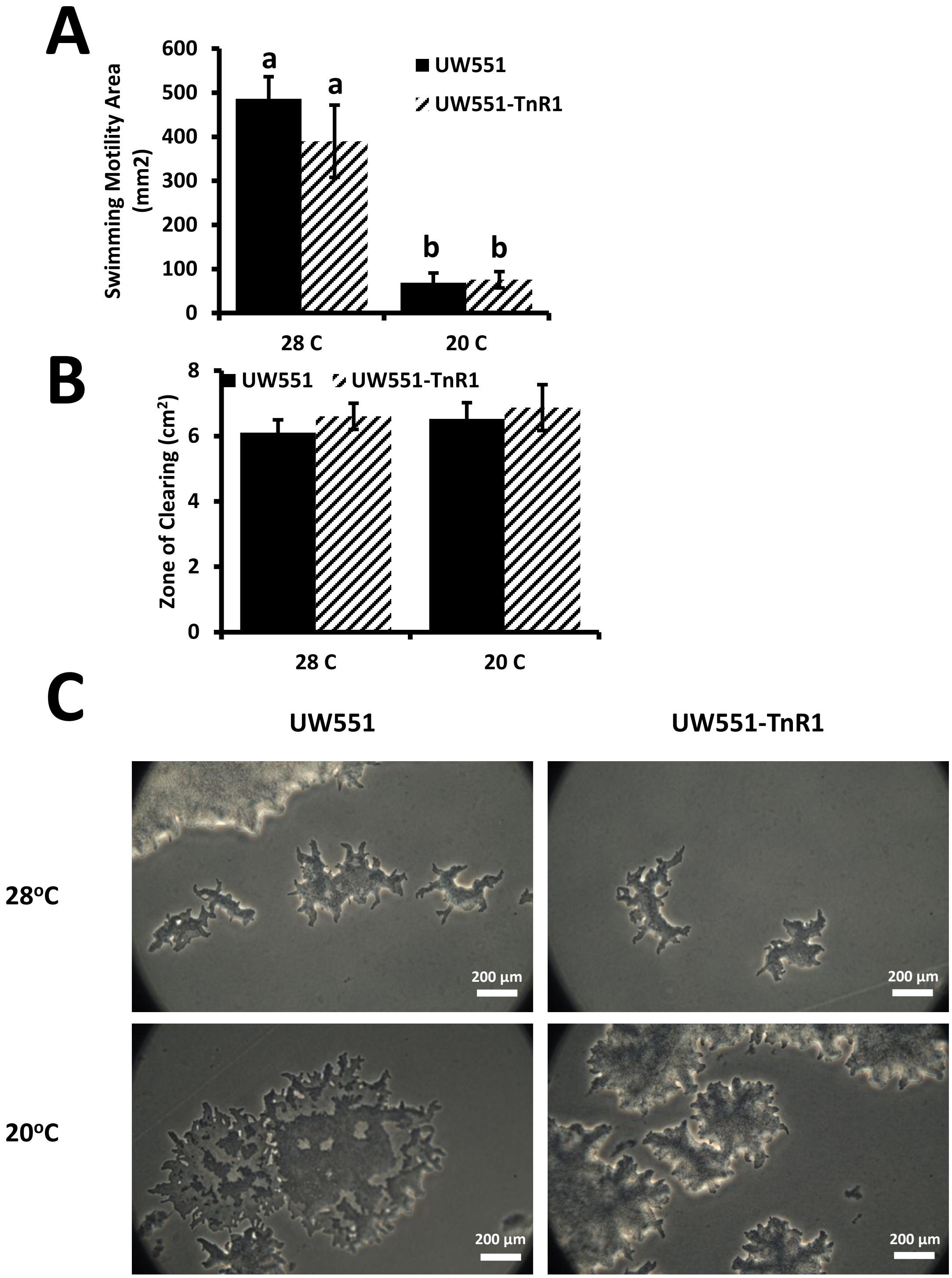

Figure S1: Comparison of virulence traits between *Ralstonia solanacearum* wild-type strain UW551 and mutant strain UW551-TnR1 tested at 28°C or 20°C. No significant differences were caused by the transposon insertion for swimming motility (A), susceptibility to exogenous hydrogen peroxide (B), and twitching motility (C).

A

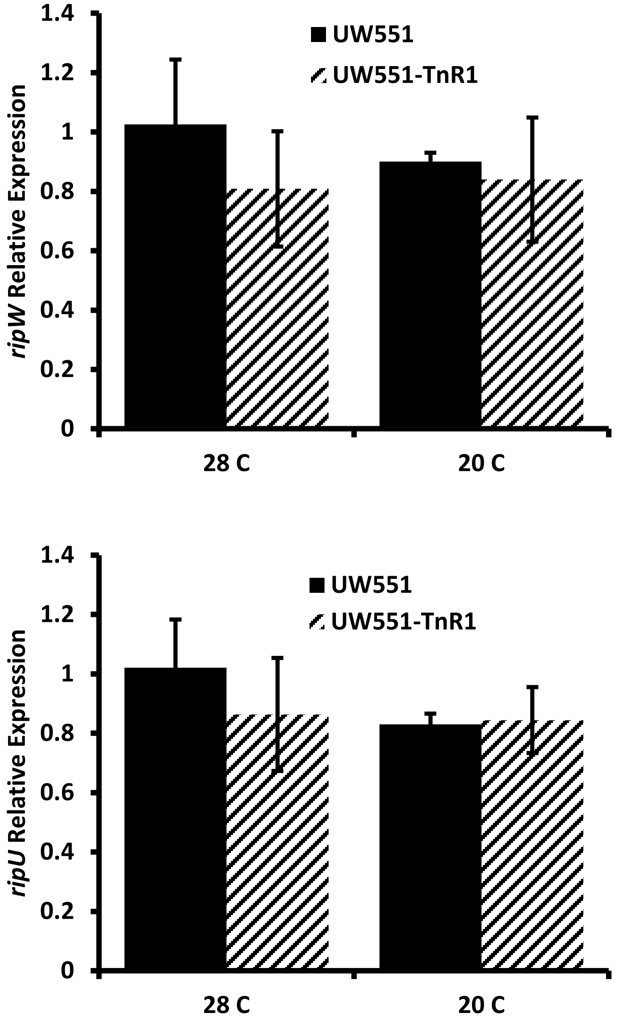

Figure S2: qRT-PCR testing of mRNA abundance of the type III effectors *ripW* (A) and *ripU* (B) grown at the indicated temperature in CPG medium shows no difference in expression based on temperature or insertion of transposon in mutant UW551-TnR1.

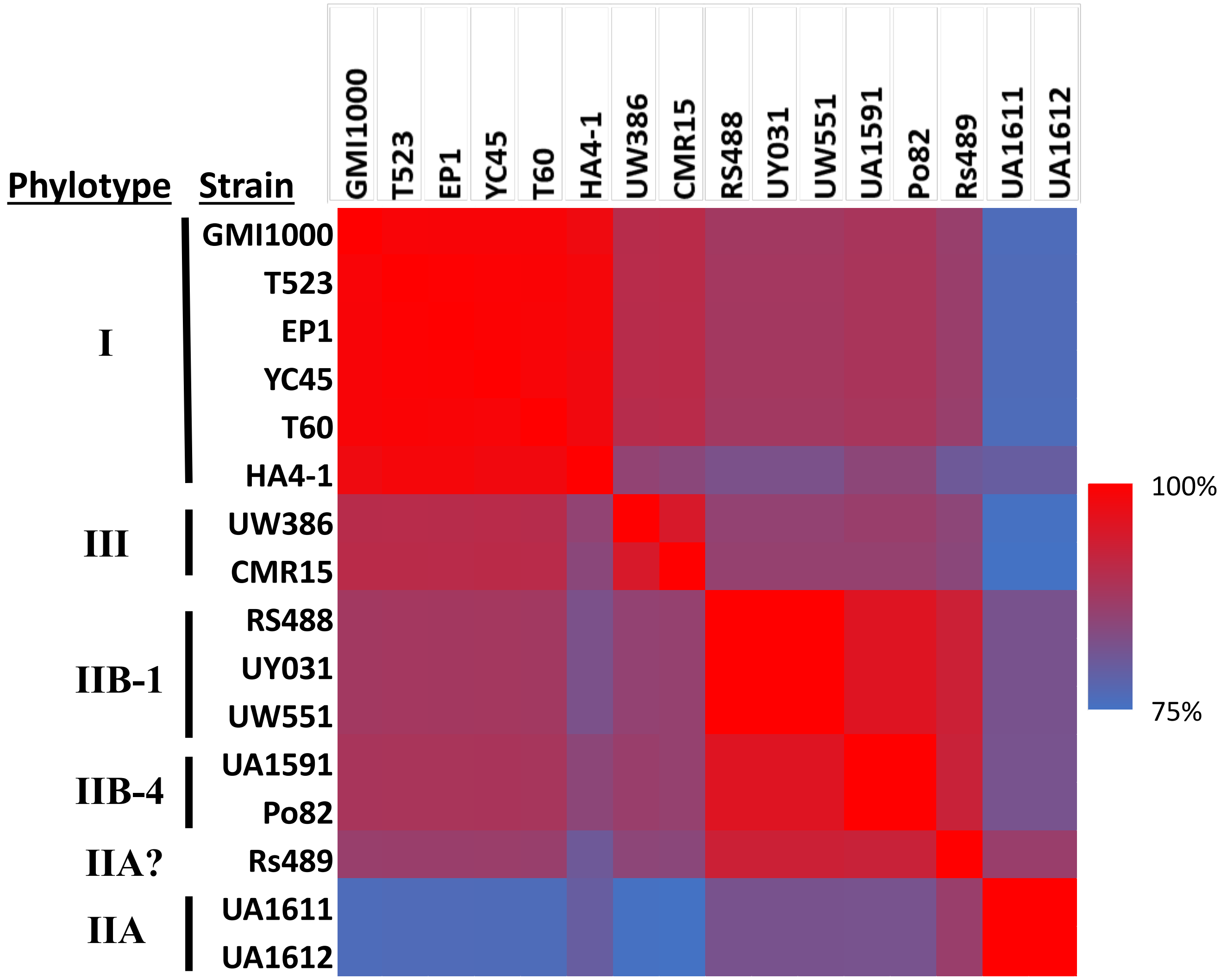

Figure S3: Heatmap representing percent identity matrix from multiple sequence alignment of type III effector RipS1 amino acids across strains representing *Ralstonia pseudosolanacearum* and *R. solanacearum* from phylotypes I, IIA, IIB, and III. Cool-virulent phylotype IIB sequevar 1 strains can be separated from other phylogenies based on RipS1 sequence.
